# Mechanistic exploration of Traditional Chinese Medicine regulation on tumor immune microenvironment in the treatment of triple-negative breast cancer: based on CiteSpace and bioinformatics analysis

**DOI:** 10.3389/fimmu.2024.1443648

**Published:** 2025-01-10

**Authors:** Dandan Feng, Dongqing Pu, Jinlu Ren, Ming Liu, Xiaohui Sun, Zhen Zhang, Jingwei Li

**Affiliations:** ^1^ Department of Breast Surgery, The First Affiliated Hospital of Zhejiang Chinese Medical University, Hangzhou, China; ^2^ The First Clinical Medical College of Shandong University of Traditional Chinese Medicine, Jinan, China; ^3^ Department of Breast and Thyroid Surgery, Affiliated Hospital of Shandong University of Traditional Chinese Medicine, Jinan, China; ^4^ Pharmaceutical college of Shandong Xiandai University, Jinan, China; ^5^ Innovation Research Institute of Traditional Chinese Medicine, Shandong University of Traditional Chinese Medicine, Jinan, China

**Keywords:** triple-negative breast cancer (TNBC), Traditional Chinese Medicine (TCM), immune regulation, CiteSpace, bioinformatics analysis, tumor microenvironments (TME)

## Abstract

**Background:**

Triple-negative breast cancer (TNBC) is a highly aggressive subtype of breast cancer, characterized by frequent recurrence, metastasis, and poor survival outcomes despite chemotherapy-based treatments. This study aims to investigate the mechanisms by which Traditional Chinese Medicine (TCM) modulates the tumor immune microenvironment in TNBC, utilizing CiteSpace and bioinformatics analysis.

**Methods:**

We employed CiteSpace to analyze treatment hotspots and key TCM formulations, followed by bioinformatics analysis to identify the main active components, targets, associated pathways, and their clinical implications in TNBC treatment.

**Results:**

CiteSpace analysis highlighted key TCM formulations, including Sanhuang Decoction. Network pharmacology identified major bioactive components such as Mutatochrome, Physcion diglucoside, Procyanidin B-5,3’-O-gallate, gallic acid-3-O-(6’-O-galloyl)-glucoside, and isomucronulatol-7,2’-di-O-glucosiole, with core targets including Mitogen-Activated Protein Kinase 1 (MAPK1), Janus Kinase 2 (JAK2), and Lymphocyte-specific protein tyrosine kinase (LCK). These targets were found to be involved in immune regulation, particularly the modulation of CD8+ and CD4+ T cells. Additionally, core targets were associated with improved recurrence-free survival (RFS) and overall survival (OS) in TNBC patients.

**Conclusion:**

The therapeutic effects of TCM in TNBC primarily involve immune modulation within the tumor microenvironment, particularly through the regulation of CD8+ and CD4+ T cells.

## 1 Introduction

Breast cancer remains one of the leading causes of cancer-related mortality among women, characterized by significant heterogeneity in its pathological features, progression, and response to treatment (1). Triple-negative breast cancer (TNBC), defined by the absence of estrogen receptors (ER), progesterone receptors (PR), and human epidermal growth factor receptor 2 (HER2), represents 12-17% of all breast cancer cases (2). This subtype is notably aggressive, exhibiting a higher propensity for invasion and metastasis compared to other breast cancer types. The primary treatment for TNBC is chemotherapy (3), but the high rates of drug resistance, recurrence, and metastasis result in poor patient outcomes. While immunotherapy has emerged as a promising treatment approach, its effectiveness in TNBC remains limited (4). These challenges underscore the urgent need for more effective therapeutic strategies.

Traditional Chinese Medicine (TCM) has demonstrated potential in treating various malignancies, including breast cancer, by improving survival rates, enhancing immune function, and alleviating symptoms (5). In TNBC, TCM plays an essential role in improving prognosis and treatment outcomes (6, 7). Network pharmacology, a method increasingly employed in TCM research, facilitates the exploration of complex disease networks and the identification of multi-target treatments (8), aligning with the holistic principles of TCM. This study integrates CiteSpace software and network pharmacology to examine current research trends in TCM for TNBC treatment, aiming to predict its mechanisms of action and validate clinical outcomes.

## 2 Materials and methods

### 2.1 Research hotspots and TCM formulations analyzed by CiteSpace

#### 2.1.1 Literature search and selection

A comprehensive search was conducted in the Web of Science (WOS) Core Collection database for literature on the treatment of TNBC with TCM from January 1, 2012, to October 21, 2022, using the following search formula:

(((TS= (Triple Negative Breast Neoplasms)) OR ALL= (triple negative breast cancer)) OR ALL= (TNBC)) AND (((TS= (traditional Chinese medicine)) OR ALL= (Chinese herbal medicine)) OR ALL= (medicinal plant)) OR ALL= (herb)).

A similar search was performed in China National Knowledge Infrastructure (CNKI) using the same criteria.

Research articles classified as REVIEW and RESEARCH were retained, while duplicates, guidelines, conference papers, news articles, and non-medical literature were excluded. Studies unrelated to the topic of TNBC were also excluded. Ultimately, 322 papers from WOS and 214 papers from CNKI were included in this study.

#### 2.1.2 Analysis method

The parameters of the analysis were set to cover the period from 2012 to 2022, with each segment representing one year. The analysis focused on keyword nodes, and the network was pruned using the Pathfinder algorithm. A “burst” value of 0.4 was applied to highlight emerging trends in TCM research for TNBC.

Keyword co-occurrence and cluster analysis were then conducted to identify research hotspots. The quality of the clustering was assessed using Q and S values. A Q value greater than 0.3 indicated the presence of a significant structure, whereas an S value greater than 0.5 suggested that the clustering was reasonable. An S value greater than 0.7 indicated a high level of confidence in the clustering results. Additionally, the strength of the clusters was analyzed, with higher strength values reflecting more prominent research frontiers and development trends in the field.

### 2.2 Analyzing the molecular mechanism through network pharmacology and molecular docking

#### 2.2.1 Identification of drug and disease targets in TNBC treatment

In the treatment of TNBC, disease targets were initially retrieved from databases such as DisGeNET (https://www.disgenet.org/), OMIM (https://www.omim.org/), TTD (https://db.idrblab.net/ttd/), Genecards (https://www.genecards.org), and CTD (https://ctdbase.org/). These targets were then integrated using the ‘Venn’ package in R (V4.3.0) for further analysis.

For identifying active compounds relevant to TNBC, we turned to the TCMSP database (https://old.tcmsp-e.com/tcmsp.php). The focus was on compounds with oral bioavailability (OB) ≥30% and drug-likeness (DL) ≥0.18. The literature was reviewed to ensure the inclusion of compounds that were appropriate for this study. To further analyze the targets of these active compounds, we utilized PharmMapper (https://lilab-ecust.cn/pharmmapper/index.html), and the gene names of the identified targets were standardized using the UniProt database (https://www.uniprot.org/).

The drug therapy targets for TNBC were determined by identifying the intersection of these two sets of targets: those derived from the active ingredients and those associated with the disease.

#### 2.2.2 Construction of protein-protein interaction (PPI) networks

Pharmacotherapeutic targets for TNBC were analyzed using the STRING database (https://string-db.org/), where only interactions with scores greater than 0.9 were selected. These targets were then imported into Cytoscape 3.7.2 software for the visualization of PPI networks. Core functional clusters were identified using the MCODE plugin, and the clusters with the highest scores were selected as key targets for further study.

#### 2.2.3 Molecular docking validation

The key targets identified from the PPI network were mapped to the corresponding components of TCM using Cytoscape. Molecular docking simulations were performed using AutoDock Vina to assess the binding affinity between the core compounds and their respective targets. A binding energy threshold of -4 kJ/mol was considered significant for stable docking.

#### 2.2.4 GO and KEGG enrichment analysis

Gene Ontology (GO) and Kyoto Encyclopedia of Genes and Genomes (KEGG) enrichment analyses were conducted using R packages such as ‘clusterProfiler’ and ‘org.Hs.eg.db’. The top 10 biological processes (BP), cellular components (CC), and molecular functions (MF) were selected based on P-values <0.01. KEGG pathway analysis was also performed to identify relevant signaling pathways.

### 2.3 Clinical data validation of key targets

#### 2.3.1 Exploring key target expression and immune infiltration in breast cancer

The core targets were analyzed for differential expression in breast cancer using the TIMER2.0 database (https://cistrome.org/TIMER2/), comparing their expression levels in breast cancer to those in normal breast tissues. Additionally, the Human Protein Atlas (HPA, https://www.proteinatlas.org/) was used to investigate the protein expression of these key targets in both cancerous and normal tissues. To further assess the relationship between these key targets and immune infiltration, the targets were also analyzed for their correlation with immune cell presence in the TNBC microenvironment, again using the TIMER2.0 database. This analysis focused on understanding how each target influences immune cell infiltration in the context of TNBC.

#### 2.3.2 Prognostic value and clinical outcome validation of key targets in TNBC

The Kaplan-Meier plotter (https://kmplot.com/) was used to evaluate the clinical relevance of core targets. Set Split patients by selecting the best cutoff, Probe set options use only JetSet best probe set. The association of key targets with overall survival (OS) and recurrence-free survival (RFS) in TNBC patients was analyzed, with ER, PR, and HER2 expression set to negative status for patient selection.

## 3 Results

### 3.1 Research hotspots and drug mining

#### 3.1.1 keyword clustering

Bibliometric analysis from the WOS and CNKI databases revealed distinct research trends in the treatment of TNBC with TCM. In the WOS database, 304 network nodes and 1040 connecting lines were identified, yielding a Q value of 0.5316 and an S value of 0.7701. In the CNKI database, 192 nodes and 303 connecting lines were found, with a Q value of 0.7146 and an S value of 0.8838. After merging overlapping clusters, the final clusters are presented in **Table 1**.

**Table 1:** TOP20 keyword clustering.

Database	Cluster Name	Size
CNKI	Quality of life	33
CNKI	Traditional Chinese medicine	26
CNKI	Breast cancer	21
CNKI	Survival rate	13
CNKI	Amorphophallus rivieri Durieu	12
CNKI	Data mining	12
CNKI	Metastasis	10
CNKI	Clinical research	8
CNKI	Resveratrol	3
WOS	Isoquinoline alkaloids	39
WOS	Breast cancer	32
WOS	mTOR	31
WOS	Experiment validation	30
WOS	EMT	29
WOS	Derivative	29
WOS	Binding	28
WOS	Post-operative recurrence	25
WOS	Triple negative breast cancer	23
WOS	Cell death	22
WOS	Mangrove	11

The analysis showed that studies in the CNKI database focused more on clinical outcomes such as quality of life, survival rates, and clinical research. In contrast, research in the WOS database leaned toward basic research topics, including mTOR, epithelial-mesenchymal transformation (EMT), and experimental validation of TCM treatments for metastatic TNBC.

Two major clusters were identified: Amorphophallus rivieri Durieu and Isoquinoline alkaloids. The Isoquinoline alkaloid cluster included studies on compounds like resveratrol, trichothecenes, cyclic adenosine monophosphate, and lignans. The Amorphophallus rivieri Durieu cluster highlighted Serpentis lupulus and Curcuma longa as key TCM ingredients.

#### 3.1.2 Keywords highlighted

Keyword highlighting analysis of the literature related to TCM treatment of TNBC in the WOS and CNKI databases was conducted, with results presented in **Table 2**. According to the ‘strength’ sorting, the types of studies in the two databases closely aligned with those found in the clustering analysis. However, in clinical-related studies, there was more emphasis on the recurrence and metastasis of TNBC, while TCM treatment methods focused primarily on strengthening the spleen. In mechanism-related studies, tumor metastasis was a central focus, along with apoptosis, cell cycle regulation, protein phosphorylation, and epithelial-mesenchymal transition. Studies also concentrated on the Nuclear Factor kappa-light-chain-enhancer of activated B cells (NFκB)-related pathway. By comprehensively analyzing both databases and extracting relevant TCM and components, the key findings included Amorphophallus rivieri Durieu, Curcuma longa, turmeric, and San Huang decoction. Bioinformatics analyses will be used to preliminarily explore the molecular mechanisms of TCM in the treatment of TNBC, incorporating these drugs into the process.

**Table 2:** TOP 25 keywords are highlighted.

Database	Keywords	strength
CNKI	Traditional Chinese medicine treatment	3.5
CNKI	Survival rate	2.22
CNKI	Prognosis	1.96
CNKI	Quality of life	1.94
CNKI	Metastasis	1.93
CNKI	Cohort studies	1.83
CNKI	Amorphophallus rivieri Durieu	1.67
CNKI	Recurrence and metastases	1.6
CNKI	Recurrence	1.52
CNKI	Chinese medicine	1.26
CNKI	Zedoary turmeric	1.11
CNKI	Fortify the spleen	1.04
CNKI	Sanhuang Decoction	1.04
WOS	Induced apoptosis	2.18
WOS	Therapy	2.07
WOS	Anticancer activity	2.04
WOS	Extract	1.73
WOS	Metastasis	1.73
WOS	NFκB	1.67
WOS	Phosphorylation	1.66
WOS	Cell cycle	1.49
WOS	Nanoparticle	1.48
WOS	In vivo	1.47
WOS	Derivative	1.46
WOS	Epithelial Mesenchymal Transition	1.39

### 3.2 Bioinformatics analysis of TCM for the treatment of TNBC

#### 3.2.1 Identification of targets in TCM for the treatment of TNBC

**Figure 1:**
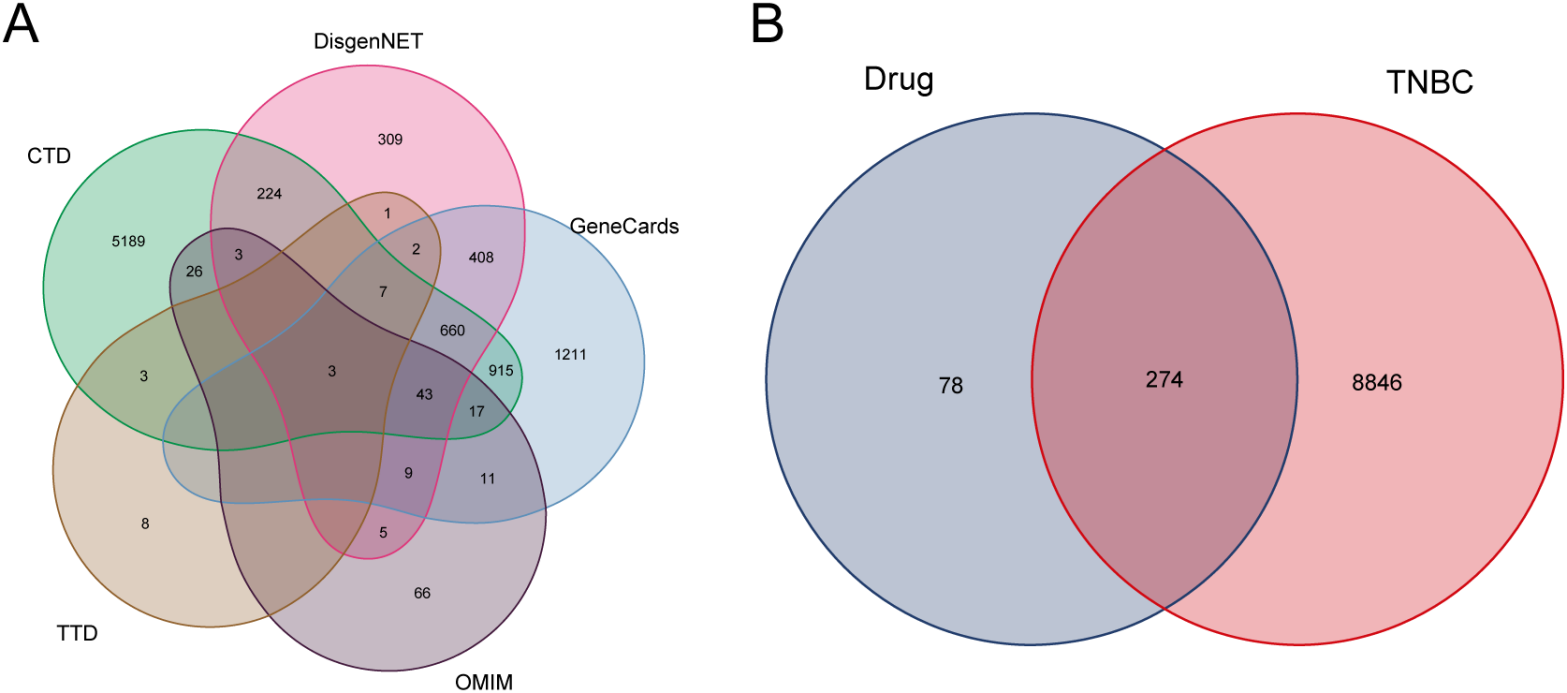
Disease targets and drug targets acting on disease. **(A)** TNBC disease targets Wayne’s diagram. **(B)** Intersection of TNBC and drug targets.

After conditional screening, the aforementioned drugs were found to contain a total of 52 active ingredients and 352 targets (**Supplementary Table S1**). Disease-related targets for TNBC were retrieved from several databases, including CTD, TTD, OMIM, DisGeNET, and GeneCards (**Figure 1A**). This process yielded a total of 9,120 disease targets, which were subsequently merged and filtered to remove redundancies (**Supplementary Table S2**). The intersection of these 9,120 disease targets and the 352 drug targets was analyzed, resulting in the identification of 274 drug targets potentially involved in the pathogenesis of TNBC (**Figure 1B**; **Supplementary Table S3**).

#### 3.2.2 PPI network analysis and extraction of key targets

**Figure 2:**
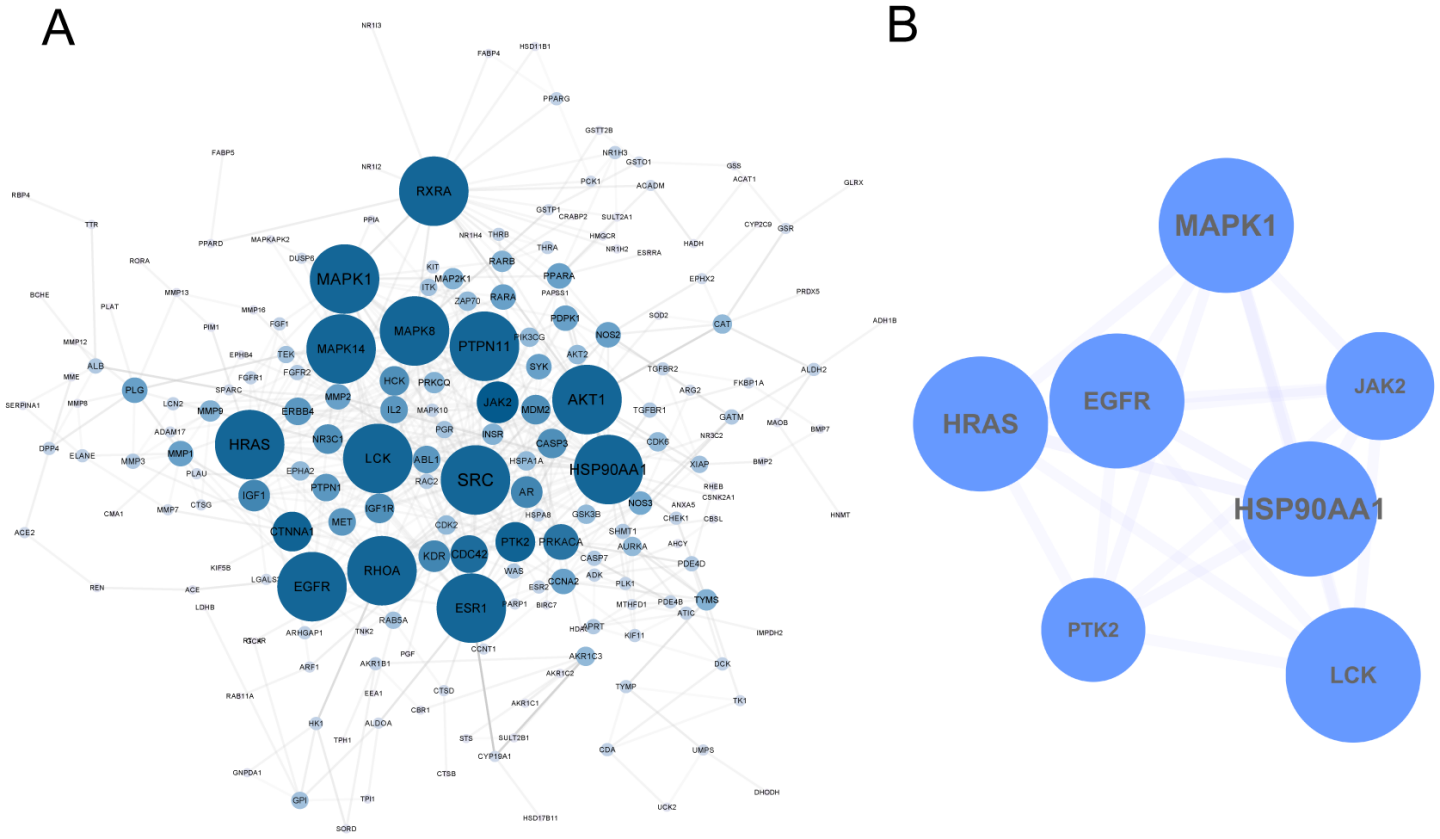
PPI Network of TCM in the Treatment of TNBC. **(A)** 274 targets of TCM for TNBC. **(B)** Key targets were extracted by MCODE.

The 274 identified targets were submitted to the STRING online database for PPI enrichment analysis. The resulting PPI network consisted of 201 nodes (targets) and 599 edges (interactions) (**Figure 2A**). To further investigate key functional clusters, the MCODE plugin within Cytoscape 3.7.2 was employed, with a node score cutoff set at 4 to ensure the selection of the most relevant clusters. This analysis resulted in the identification of a single cluster comprising seven targets, which were ranked in descending order based on their degree centrality: Heat Shock Protein 90 Alpha Family Class A Member 1 (HSP90AA1), Mitogen-Activated Protein Kinase 1 (MAPK1), Harvey Rat Sarcoma Viral Oncogene Homolog (HRAS), Epidermal Growth Factor Receptor (EGFR), Lymphocyte-specific protein tyrosine kinase (LCK), Protein Tyrosine Kinase 2 (PTK2), and Janus Kinase 2 (JAK2) (**Figure 2B**). These seven targets were subsequently defined as the key targets of this analysis, reflecting their pivotal roles in the biological network under investigation.

#### 3.2.3 The ‘core ingredient-target’ network of TCM for TNBC treatment

**Figure 3:**
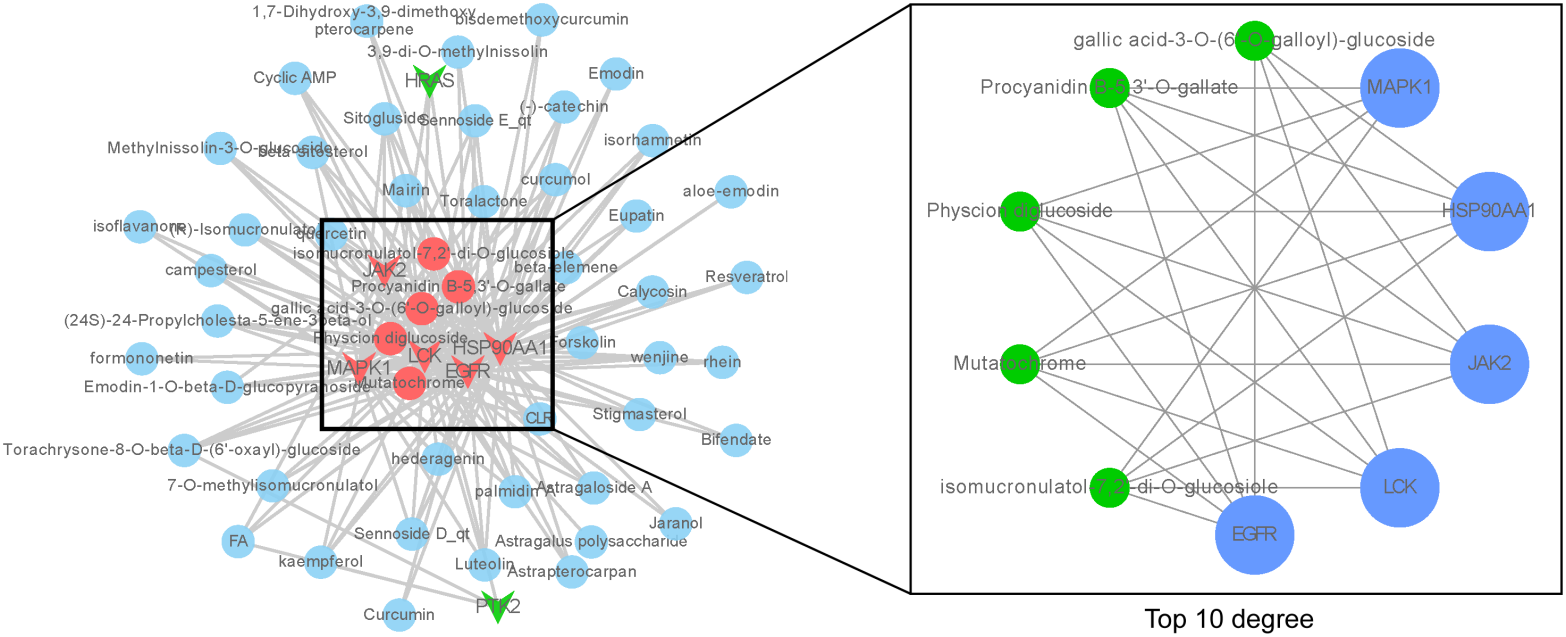
‘Core ingredient-target’ network. The left figure shows the ‘core ingredient-target network’, and the right figure shows the ingredient-target network with top 10 degree values.

To further elucidate the active ingredients interacting with the seven key targets, reverse mapping was performed to construct the “key ingredient-target” network, which consisted of 50 active ingredients, seven targets, and 179 edges, as depicted in **Figure 3**. Initial network analysis was conducted using the Network Analyzer plugin in Cytoscape software. Subsequently, the top ten nodes were identified using the cytoHubba plugin, which included five core active ingredients: Mutatochrome, Physcion diglucoside, Procyanidin B-5,3’-O-gallate, gallic acid-3-O-(6’-O-galloyl)-glucoside, and isomucronulatol-7,2’-di-O-glucosiole. The first four ingredients belong to rhubarb, while the last ingredient, isomucronulatol-7,2’-di-O-glucosiole, belongs to astragalus. The analysis also revealed five core targets: MAPK1, LCK, JAK2, HSP90AA1, and EGFR, which are integral to the biological processes involved in TNBC. The interactions between these five ingredients and the five targets are defined as the ‘Core ingredient-target’ network.

#### 3.2.4 Molecular docking validation of ‘Core ingredient-target’

**Figure 4:**
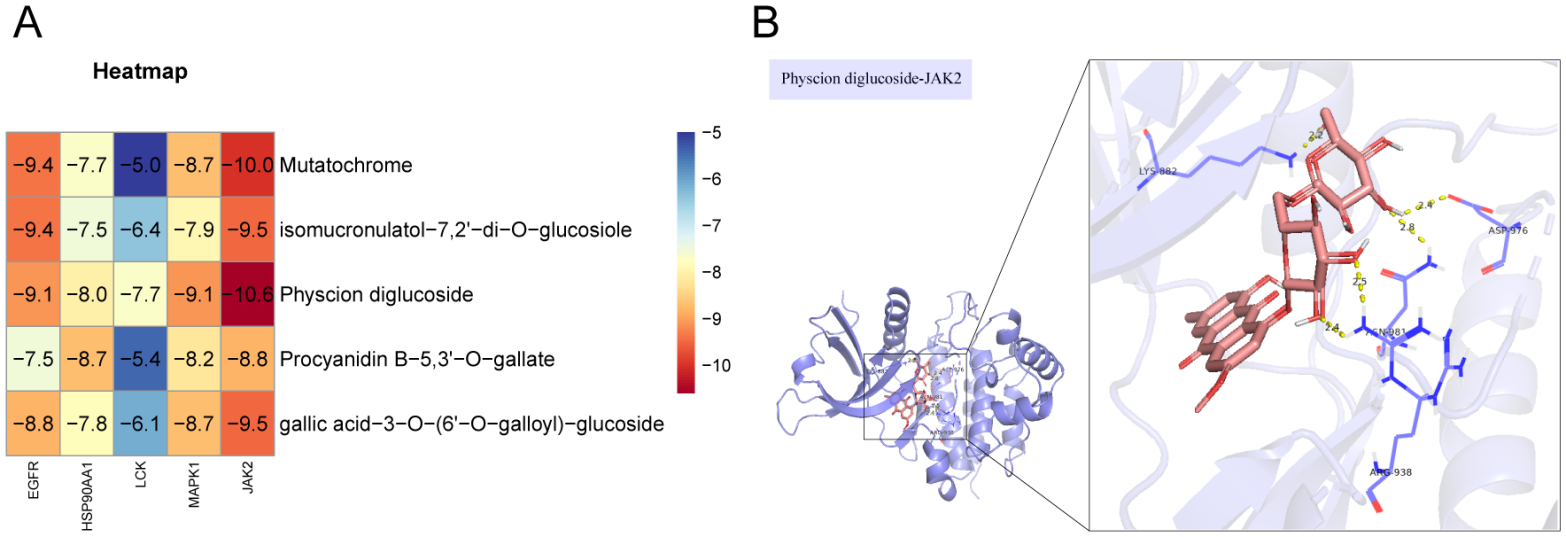
Molecular docking validation. **(A)** In molecular docking thermogram of component targets, the redder the color, the lower the binding energy, which represents a higher binding strength. **(B)** Docking pattern diagram of Physcion diglucoside and JAK2.

To further validate the effect of the ingredients in the ‘Core ingredient-target’ network on the targets, molecular docking studies were performed. Five ingredients were able to bind to each of the five targets, with binding energies less than -4.7 kJ/mol (**Figure 4A**), suggesting that all docking interactions are valid. Moreover, lower binding energies indicate more stable interactions. As shown in **Figure 4A**, JAK2 exhibited strong docking interactions with all small molecules, with the most stable binding observed between JAK2 and Physcion diglucoside, which had a binding energy of -10.6 kJ/mol. The specific docking mode is shown in **Figure 4B**, where eight hydrogen bonds were formed between JAK2 and Physcion diglucoside, with the docked amino acid residues being LYS-882, ASP-976, ARG-938, and AGN-981. These results suggest that Physcion diglucoside may be an effective ingredient for TNBC treatment, with JAK2 potentially playing a key role in the therapeutic process.

#### 3.2.5 GO enrichment analysis

**Figure 5:**
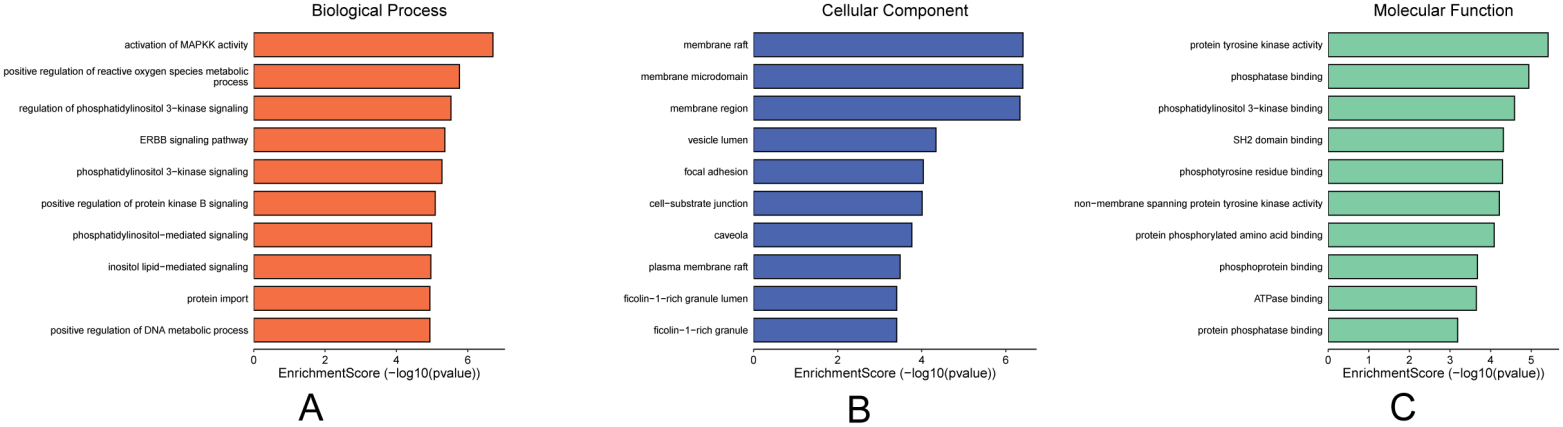
Plot of GO enrichment analysis of core targets. **(A)** GOBP enrichment analysis, **(B)** GOCC enrichment analysis, **(C)** GOMF enrichment analysis, importance in order of p-value.

Molecular docking validation confirmed that the components in the “Core Ingredient-Target” network bind effectively to the targets. This led us to explore the biological processes regulated by these components. Therefore, we performed GO enrichment analysis on the five targets in this network, examining GOBP, GOCC, and GOMF. All results were ranked according to P-values, with the top ten GOBP terms including four related to immune responses: activation of immune response, immune response-activating cell surface receptor signaling pathway, and immune response-activating signal transduction (**Figure 5A**). The highest-ranked GOCC terms were membrane rafts, membrane microdomains, focal adhesion, cell-matrix junctions, vesicle lumen, caveolae, and extrinsic components of the cytoplasmic side of the plasma membrane (**Figure 5B**). The top ten GOMF terms included phosphatase binding, protein tyrosine kinase activity, protein phosphatase binding, SH2 domain binding, non-membrane spanning protein tyrosine kinase activity, protein C-terminal binding, phosphotyrosine 3-kinase binding, phosphotyrosine residue binding, and ATPase binding (**Figure 5C**). These findings suggest that the core components likely play a significant role in the immune pathways of TNBC, primarily through the activation of phosphatases and related mechanisms.

#### 3.2.6 KEGG enrichment analysis

**Figure 6:**
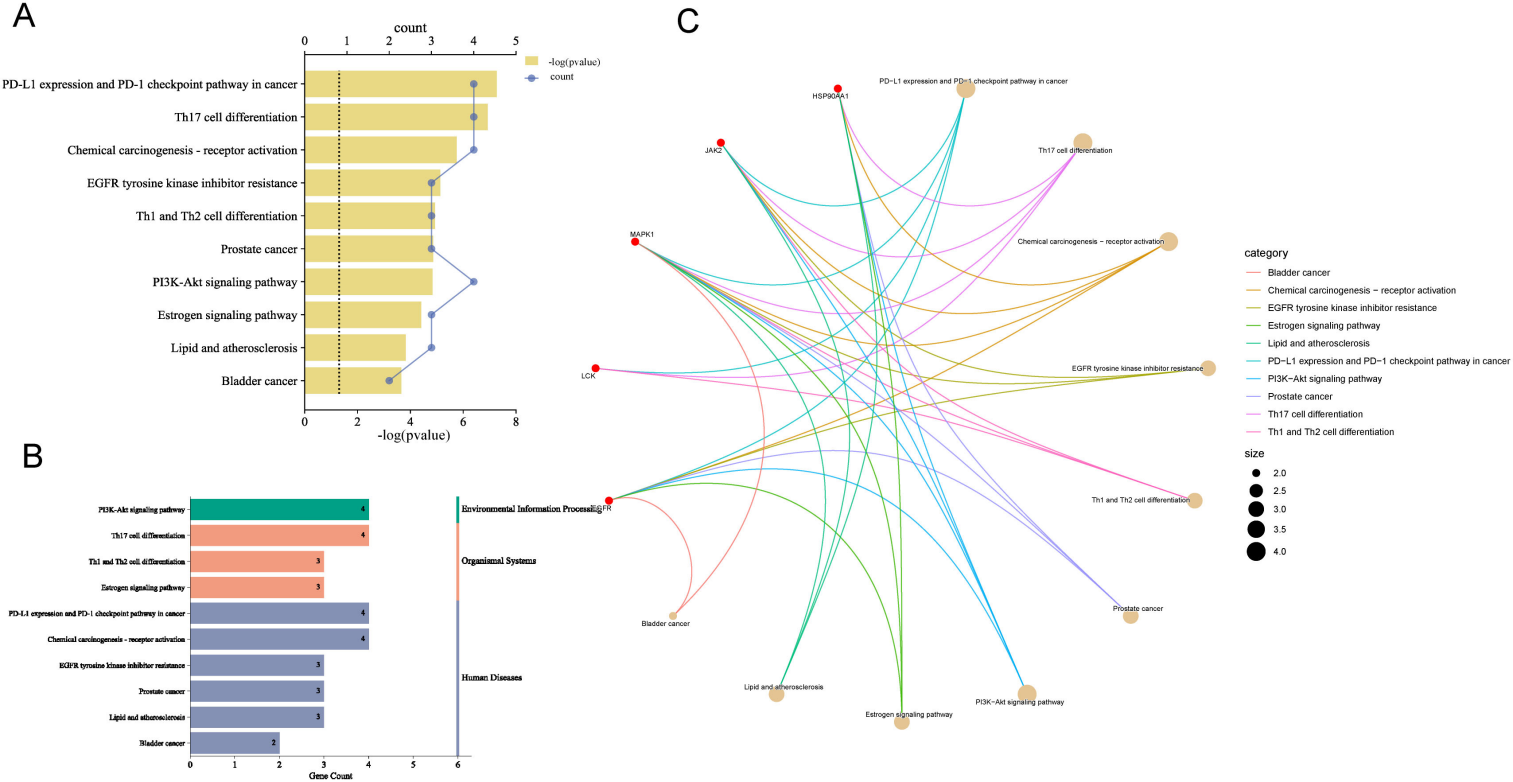
Enrichment analysis of KEGG pathways. **(A)** Pathways with top ten P-values, the smaller the P-value the more important. **(B)** Secondary analysis of pathways. **(C)** Chordal plot of pathways and their targets, node size is proportional to the number of items connected.

The top 10 KEGG pathways were visualized according to P-value ordering (**Figure 6A**), which included pathways such as PD-L1 expression and PD-1 checkpoint pathway in cancer, PI3K-Akt signaling pathway, chemical carcinogenesis-receptor activation, lipid and atherosclerosis, EGFR-Thyrosine kinase inhibitor research, thyrosine kinase inhibitor resistance, Th1 and Th2 cell differentiation, prostate cancer, Th17 cell differentiation, growth hormone synthesis, estrogen signaling pathway, bladder cancer, and four pathways related to immune cell regulation. These pathways were then analyzed through secondary classification, as depicted in **Figure 6B**, with one pathway classified under environmental information processing, two under cellular processes, three under organic systems, and six under human diseases. For further analysis, the pathways and their associated targets were visualized, as shown in **Figure 6C**, where the immune-related targets identified include LCK, MAPK1, and JAK2.

The findings suggest that the pathways regulated by the core ingredients are primarily immune-related, with the PD-L1 expression and PD-1 checkpoint pathway being the most prominent and highly associated with TNBC. The involvement of JAK2, MAPK, LCK, and EGFR in this pathway aligns with the GOBP analysis, further supporting the strong association of the “Core Ingredient-Target” network with immunotherapy in TNBC.

### 3.3 Expression of core targets in breast cancer

**Figure 7:**
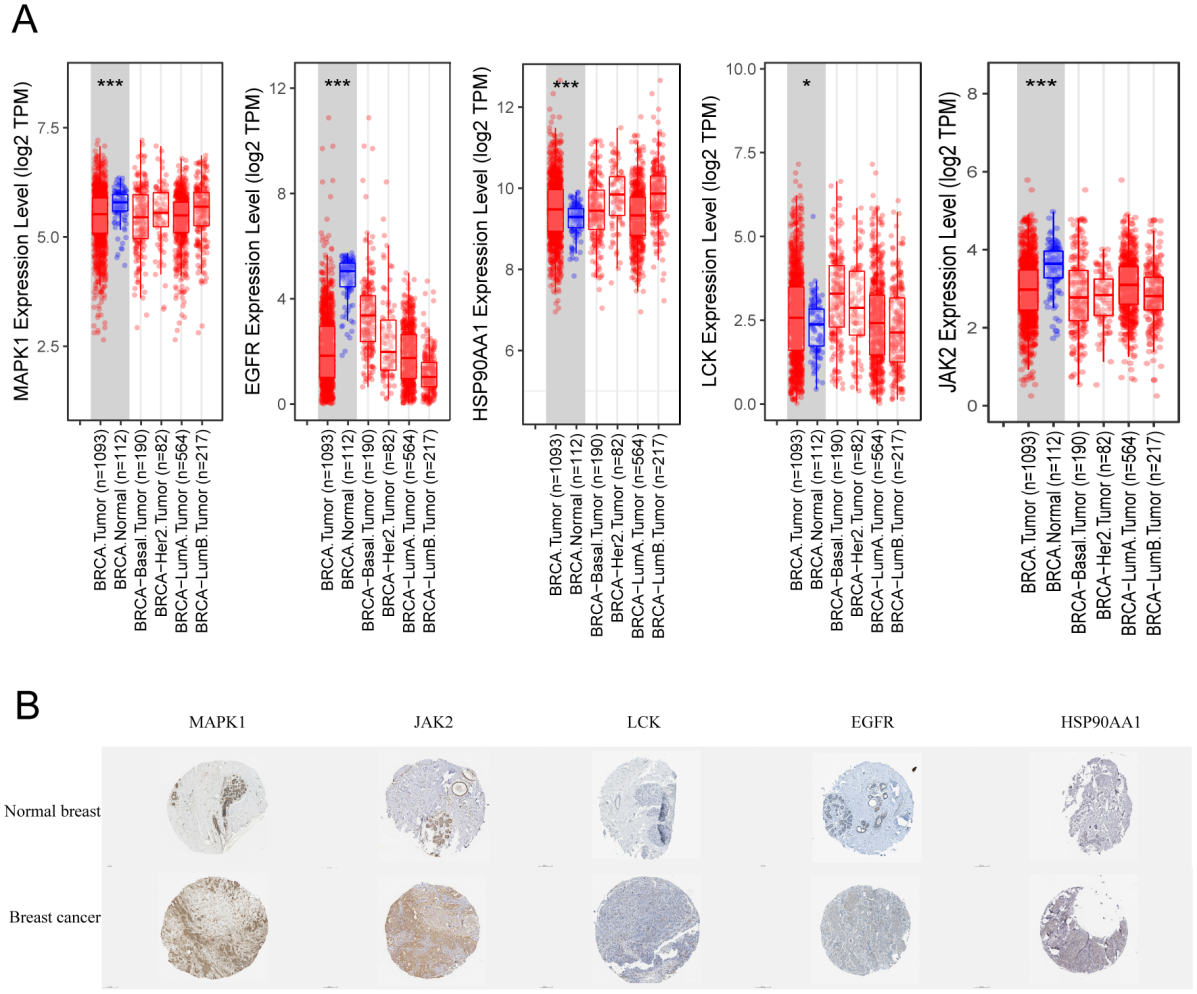
Expression of key targets in breast cancer. **(A)** Box line plot of expression differences in different types of breast cancer. **(B)** Immunohistochemistry of pathological tissues from breast cancer patients. * p<0.05, ***p<0.001.

To further validate the RNA expression levels of the five targets MAPK1, LCK, JAK2, HSP90AA1, and EGFR, the TIMER2.0 online database was utilized. The analysis indicated that all five targets exhibit specific expression in breast cancer. Among these, MAPK1, EGFR, and JAK2 showed lower expression levels, while HSP90AA1 and LCK were more highly expressed (**Figure 7A**). Although the database does not provide comparisons across breast cancer subtypes, the box plots indicate that the expression levels of these targets in BRCA-basal and BRCA-tumor samples are similar. Based on this, we infer that these targets are specifically expressed in TNBC. Notably, LCK showed significantly higher expression in TNBC compared to normal tissues and other breast cancer subtypes, whereas MAPK1 and JAK2 exhibited much lower expression in TNBC compared to normal tissues and other breast cancer subtypes.

Additionally, immunohistochemical results from the HPA online database were examined for breast cancer patients. As shown in **Figure 7B**, MAPK1 and JAK2 demonstrated the strongest specificity and the best staining in breast cancer tissues. In contrast, LCK was almost undetectable, while EGFR and HSP90AA1 showed weak positive expression. It is worth noting that the transcriptional expression levels of JAK2 and MAPK1 in breast cancer tissues differ from their protein expression levels.

### 3.4 Relationship between core targets and immune infiltration

**Figure 8:**
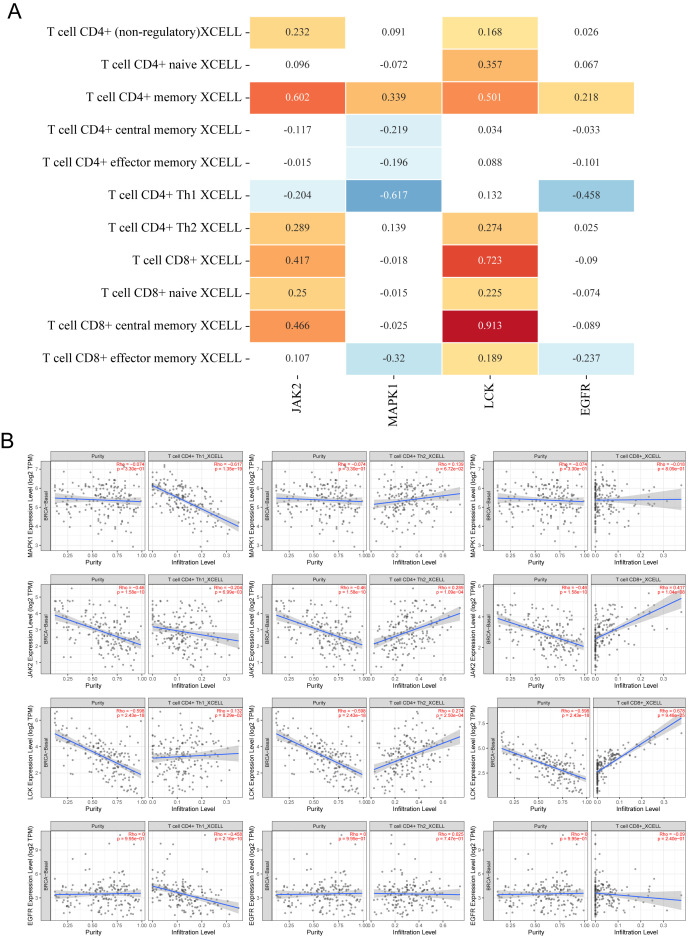
Relationship between core targets and immune cell infiltration. **(A)** Heatmap of correlation between 4 targets and infiltration of different CD4^+^ T cells and CD8^+^ T cells, the numbers in the figure are Spearman’s rho values P, yellow is P < 0.05, p > 0 is a positive correlation; blue is P < 0.05, p < 0 is a negative correlation, p > 0.05 is meaningless. **(B)** Scatterplot of correlation between different targets and infiltration of Th1, Th2, and CD8^+^ T cell infiltration scatter plots.

Since both KEGG and GOBP enrichment analyses point toward tumor immune infiltration, it suggests that the core components may intervene in TNBC through immune pathways. To validate this hypothesis, we analyzed the immune infiltration correlation of four immune-related targets (JAK2, MAPK1, EGFR, and LCK) using the TIMER2.0 website. As Th17 cells were not included in the database, we focused on analyzing the expression of PD-L1 expression and PD-1 checkpoint pathway in cancer, and Th1 and Th2 cell differentiation. As shown in **Figure 8A**, the relationship between pathway-regulated targets and immune infiltration of different types of CD4+ and CD8+ T cells was analyzed using the XCELL algorithm. LCK and JAK2 were generally positively correlated with both CD8+ and CD4+ T cells, while EGFR and MAPK1 showed mostly negative correlations. As shown in **Figure 8B**, when JAK2, EGFR, and MAPK1 had low expression, the infiltration of Th1 cells was elevated. When JAK2 and LCK were highly expressed, the infiltration levels of Th2 and CD8+ T cells significantly increased. Based on the above results, we observed that only JAK2 showed a significant regulatory effect on all three immune cell types.

### 3.5 Modulation of prognosis in TNBC by core targets

**Figure 9:**
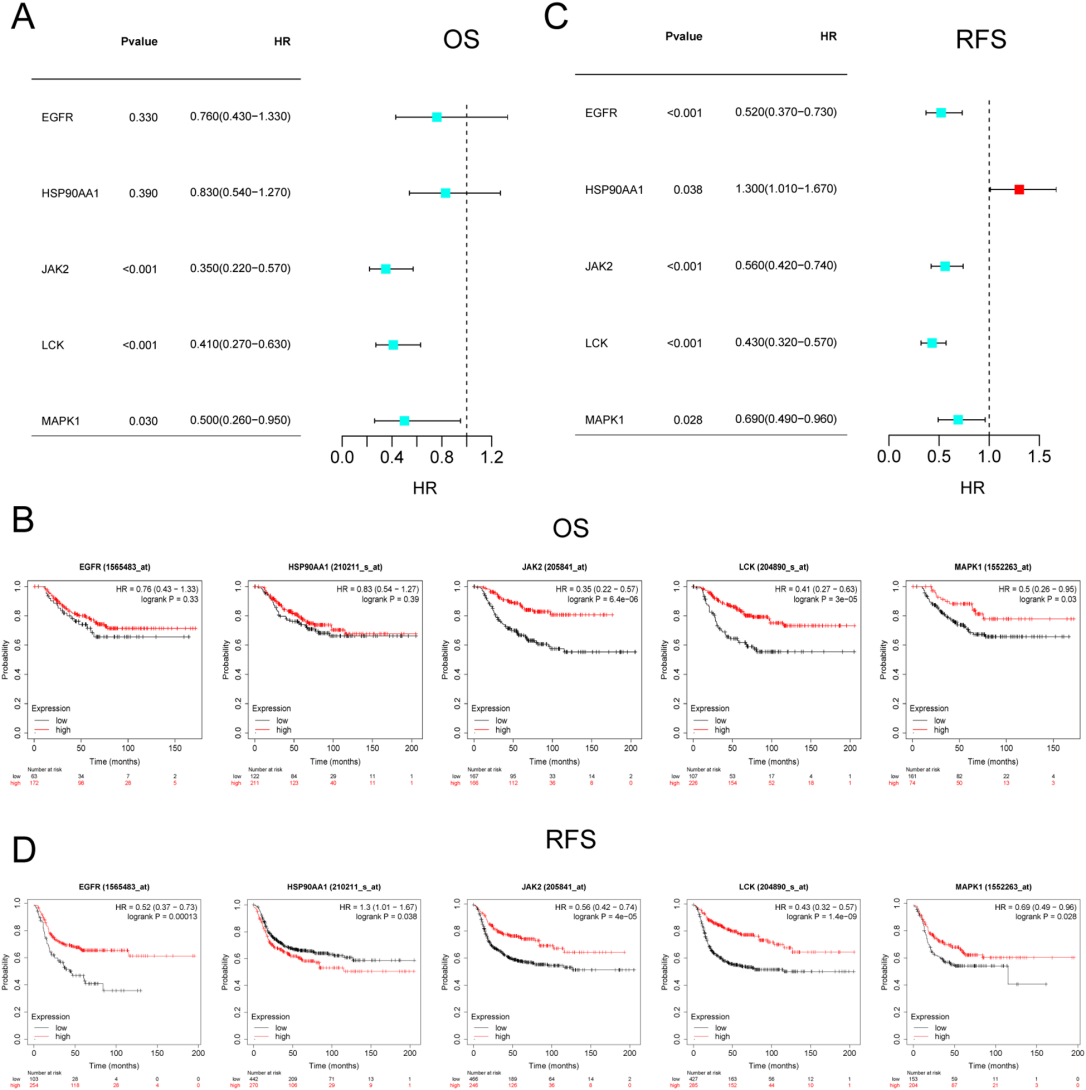
Survival analysis of core targets. **(A, B)** KM plots and forest plots of core targets versus OS. **(C, D)** KM plots and forest plots of core targets versus RFS. P < 0.05 was considered significant.

Since the core targets regulate immune cell infiltration in TNBC, the next question we sought to address is whether these targets influence the survival of TNBC patients. The impact of JAK2, MAPK1, EGFR, HSP90AA1, and LCK on OS and RFS in TNBC patients was validated using the Kaplan-Meier Plotter online tool. The results indicated that JAK2, MAPK1, and LCK were significantly associated with OS in TNBC patients (P < 0.05) (**Figure 9A**), with increasing gene expression levels corresponding to higher OS (**Figure 9B**). Furthermore, all five targets were found to be correlated with RFS in TNBC patients (P < 0.05) (**Figure 9C**), with RFS survival rates positively correlating with gene expression levels (**Figure 9D**). The targets that were correlated with both OS and RFS included JAK2, LCK, and MAPK1, further suggesting that the core components may intervene in the progression of TNBC by modulating these targets.

## 4 Discussion

In the treatment of TNBC, TCM is commonly used as an adjunct to chemotherapy. TCM aims to alleviate the side effects of chemotherapy, reduce toxicity, overcome drug resistance, and enhance the overall therapeutic efficacy, ultimately improving clinical outcomes (9, 10). The therapeutic principle of TCM for TNBC involves ‘reinforce the healthy qi and eliminate the pathogenic factors’, which regulates the immune system to suppress tumor progression and metastasis. This approach is conceptually aligned with the precision medicine strategies used in current immunotherapies for TNBC, particularly with the use of PD-1 and PD-L1 inhibitors such as atezolizumab and pembrolizumab, which have been approved for both early and advanced TNBC (11). However, immune non-response remains a significant challenge in immunotherapy, underscoring the urgent need for the development of new immune agents. Several TCM formulations, including Liuwei Dihuang Wan, Sanhuang Decoction, Sanhuang Xiexin Decoction, and Yougui Wan, have shown promising results in clinical trials for TNBC, demonstrating their potential to enhance immune function and inhibit tumor progression (12–15). Despite these clinical successes, the precise molecular mechanisms by which TCM modulates the tumor immune microenvironment remain unclear. This study combines bibliometric and bioinformatics analyses to identify key drugs and research trends in TCM for TNBC treatment and to explore the underlying molecular mechanisms. Our findings offer new insights that may contribute to both the treatment and future research on TNBC, providing a foundation for more targeted and effective therapeutic strategies.

Through bibliometric and bioinformatics analyses, the core components currently associated with TCM in the treatment of TNBC include Mutatochrome, Physcion diglucoside, Procyanidin B-5,3’-O-gallate, gallic acid-3-O-(6’-O-galloyl)-glucoside, and isomucronulatol-7,2’-di-O-glucosiole. Network mapping analysis reveals that the first four components are derived from rhubarb, while the last is sourced from astragalus. Studies indicate that water extracts of rhubarb contain various bioactive compounds, such as anthraquinones, flavonoids, quinones, gallic acid esters, and tannins, all of which exhibit diverse pharmacological effects, including antioxidation, anti-inflammation, antimicrobial activity, and immune modulation (16). Among these, anthraquinones like rhapontigenin and rhaponticin are known to regulate immune responses, reduce prostaglandin E2 production during inflammation, and inhibit the release of inflammatory cytokines (e.g., IL-2, TNFα) and MMP9 (17). Additionally, rheumemodin has been shown to significantly suppress T-cell and B-cell proliferation, while also reducing cytokine expression, demonstrating its immune-modulatory activity (18). The core component Physcion diglucoside, belonging to the anthraquinone class, is recognized for its prominent anti-inflammatory, anti-tumor, and antioxidant activities (19). Similarly, gallic acid-3-O-(6’-O-galloyl)-glucoside, a tannin compound, has been found to exhibit anticancer properties by inducing ROS generation in cancer cells, which triggers the activation of caspases 3, 8, and 9, the release of cytochrome c, and a loss of mitochondrial potential, ultimately leading to apoptosis in cancer cells (20). The polyphenolic compound Procyanidin B-5,3’-O-gallate also demonstrates potent antioxidant effects and effectively inhibits cancer cell proliferation (21, 22). Mutatochrome, a carotenoid, has shown potential in inhibiting cancer cell proliferation (23). Astragalus is well-known for its immune-regulatory properties, particularly through its primary component, astragalus polysaccharide, which enhances the proliferation of T and B lymphocytes in tumor-bearing mice, promotes IL-2 production by spleen cells, and restores normal levels of IL-2, thereby improving cellular immune function and contributing to anti-tumor effects (24). The flavonoid isomucronulatol-7,2’-di-O-glucosiole, isolated from Astragalus, inhibits IL-12 production induced by LPS *in vitro*, demonstrating significant anti-inflammatory activity (25). These findings suggest that the regulatory effects of the five core components on tumors are closely associated with immune and inflammatory responses. This is consistent with the results predicted by our GOBP analysis, which highlights the activation of immune response, immune response-activating cell surface receptor signaling pathway, and immune response-activating signal transduction as key enriched pathways, all of which point to immune modulation. Moreover, several pathways identified in the KEGG enrichment analysis also directly implicate immune regulation, further supporting the central role of immune modulation in the anti-tumor effects of these TCM components.

Tumor immune modulation enables cancer cells to evade immune surveillance and clearance, thereby promoting their survival and growth. Intervening in tumor immune regulation is particularly critical for cancers like TNBC, where targeted therapies are limited. Among the top pathways identified in KEGG enrichment analysis is the PD-L1 expression and PD-1 checkpoint pathway in cancer. Immune checkpoints are fundamental to current immunotherapy for TNBC, and the blockade of PD-1 and PD-L1 represents a key strategy to prevent tumor immune evasion (26). Additionally, immune cell therapies are increasingly being applied in the treatment of solid tumors. Our findings suggest that, beyond the PD-1/PD-L1 signaling pathway, the differentiation of Th17, Th1, and Th2 cells is closely associated with TCM in the treatment of TNBC. These cell types, all derived from CD4+ T cells under specific stimuli, secrete distinct cytokines and play different roles in immune responses. In TNBC, Th1 responses are generally linked to a favorable prognosis. Th1 cells produce IFN-γ, which enhances the expression of MHC I on tumor cells, thereby increasing the ability of CD8+ T cells to recognize and eliminate tumor cells (27, 28). In contrast, Th2 and Th17 responses are typically associated with tumor progression. Cytokines secreted by Th2 cells, such as IL-4 and IL-13, disrupt the balance between Th1 and Th2, shifting the immune response from Th1 to Th2, thereby promoting tumor growth (29). Th17 cells produce IL-17A, which facilitates cancer cell migration, invasion, and stemness through the STAT3/NF-κB/Notch1 signaling pathway, while also recruiting MDSC cells in the TNBC tumor microenvironment, further enhancing tumor progression (30, 31). Thus, Th2 and Th17 responses are generally associated with poor prognosis in TNBC. Unlike CD4+ T cells, which indirectly regulate other immune cells (such as CD8+ T cells, B cells, and macrophages) to help eliminate tumor cells, CD8+ T cells directly attack and destroy tumor cells. However, CD8+ T cell exhaustion is a major obstacle to their anti-tumor function (32). In addition to PD-1/PD-L1, it is equally crucial to explore other pathways that inhibit CD8+ T cell exhaustion, promote Th1 differentiation, and suppress the differentiation of Th2 and Th17 cells.

In this study, the core components regulate five key targets: MAPK1, LCK, JAK2, HSP90AA1, and EGFR, all of which are uniquely expressed in breast cancer. Among these, the gene expression levels of JAK2 and MAPK1 are lower in breast cancer tumors compared to normal breast tissue, yet their protein expression is elevated. Several factors contribute to this phenomenon, including the decoupling of gene and protein expression, post-transcriptional regulatory mechanisms, the impact of epigenetic modifications, and discrepancies in protein stability and degradation rates (33–35). Survival analysis revealed that the expression of JAK2, LCK, and MAPK1 is positively correlated with OS and RFS in TNBC patients. Furthermore, the results suggest that JAK2 and LCK expression is positively correlated with CD8+ T cell infiltration, which may explain why high expression of these proteins promotes both OS and RFS in TNBC patients. By enhancing CD8+ T cell infiltration, these targets facilitate tumor cell destruction and prevent recurrence, ultimately prolonging survival. Current research indicates that JAK2 drives proliferation, metabolism, immunity, inflammation, and malignancy through various cytokines, interferons, and growth factors within the JAK-STAT3 pathway (36). Similarly, the MAPK1 signaling pathway plays a role in regulating dendritic cells, CD4+ T cells, and tumor antigen-specific CD8+ T cells (37). LCK, a critical player in T cell activation, influences T cell regulation and the infiltration of CD8+ T cells through its interactions with other proteins (38). Additionally, research suggests that JAK2 amplification during neoadjuvant chemotherapy is associated with metastatic spread, and targeting JAK2 alone has been shown to be effective in treating TNBC (39). EGFR overexpression, found in more than 50% of TNBC cases, is recognized as a key driver of TNBC progression (40), while HSP90AA1 upregulation independently increases the risk of TNBC recurrence and poor prognosis (41). These findings reinforce the credibility of our research. Moreover, favorable molecular docking results further suggest that the core components interact effectively with these key targets, indicating that the five core components, including Mutatochrome, may enhance CD8+ T cell immune infiltration through modulation of JAK2, LCK, and MAPK1, thereby extending the survival of TNBC patients. These insights underscore the potential of TCM as a therapeutic approach for TNBC.

Current research on TCM in the treatment of TNBC primarily focuses on clinical studies, which have demonstrated significant therapeutic efficacy. However, most of these studies involve TCM in combination with conventional chemotherapy. For instance, the combination of Nüzhen Lian Gui Bai Shao Decoction with adjuvant chemotherapy has been shown to significantly reduce tumor marker expression in postoperative TNBC patients, modulate immune function, and mitigate adverse reactions (42). The use of Sanhuang Decoction alone for six months significantly improves inflammation factor levels, reduces chronic oxidative stress, and enhances immune function in TNBC patients (43). Similarly, Yanghe Decoction, when combined with the GT chemotherapy regimen, effectively regulates immune function in stage IV TNBC, upregulating Th17 levels and downregulating Treg levels (44). Additionally, combining Senqi Fuzheng Injection with TAC chemotherapy for TNBC significantly increases the levels of NK cells, CD3+ T cells, CD4+ T cells, and CD4+/CD8+ T cell ratios, contributing to reduced toxicity and improved therapeutic efficacy (45). These studies not only complement but also reinforce the findings of our research. Nevertheless, there are several limitations in this study, including the absence of experimental validation for the molecular mechanisms involved and a lack of clinical data on the efficacy of the drugs. Additionally, due to the constraints of our search criteria, the selected studies may carry some bias and might have excluded important monomeric drugs. Moreover, the inherent diversity of chemical components and targets in TCM presents challenges in fully understanding its therapeutic effects. Single components and targets cannot comprehensively account for the efficacy and mechanisms of action of entire TCM formulas. This represents a fundamental limitation in current TCM mechanism research. Despite these challenges, the methods used in this study offer valuable insights that could inform future investigations. As for whether the five core components, such as Mutatochrome, can promote CD8+ T cell immune infiltration through key targets like JAK2, LCK, and MAPK1, thereby extending the survival of TNBC patients, we plan to conduct further laboratory experiments and clinical studies to validate and analyze this hypothesis.

## 5 Conclusion

In conclusion, this study investigates the molecular mechanisms underlying the therapeutic effects of TCM in the treatment of TNBC by integrating bibliometric and bioinformatics analyses. Five core components, including Mutatochrome, were identified to regulate key targets such as JAK2, LCK, and MAPK1, with the ability to modulate the PD-1/PD-L1 signaling pathway. Furthermore, these components influence the immune infiltration of CD8+ T and CD4+ T cells, thereby impacting the OS and RFS of TNBC patients. These findings provide novel insights and valuable guidance for advancing TCM-based therapeutic strategies in TNBC treatment.

## Data Availability

The datasets presented in this study can be found in online repositories. The names of the repository/repositories and accession number(s) can be found in the article/**Supplementary Material**.
